# Trajectory modeling of endothelial-to-mesenchymal transition reveals galectin-3 as a mediator in pulmonary fibrosis

**DOI:** 10.1038/s41419-021-03603-0

**Published:** 2021-03-26

**Authors:** Wangyue Jia, Zhaoyan Wang, Ceshu Gao, Jian Wu, Qiong Wu

**Affiliations:** 1 MOE Key Laboratory of Bioinformatics, School of Life Sciences, Tsinghua University, Beijing, China; 2 Center for Synthetic and Systems Biology, School of Life Sciences, Tsinghua University, Beijing, China; 3 Tsinghua-Peking Center for Life Sciences, Tsinghua University, Beijing, China; 4 Department of Neurology, Beijing Tsinghua Changgung Hospital, School of Clinical Medicine, Tsinghua University, Beijing, China

**Keywords:** Mechanisms of disease, Transcriptomics

## Abstract

The endothelial-to-mesenchymal transition (EndMT) is an important source of fibrotic cells in idiopathic pulmonary fibrosis (IPF). However, how endothelial cells (ECs) are activated and how EndMT impact IPF remain largely elusive. Here, we use unsupervised pseudotemporal analysis to recognize the heterogeneity of ECs and reconstruct EndMT trajectory of bleomycin (BLM)-treated *Tie2*^*creER/+*^*;Rosa26*^*tdTomato/+*^ IPF mice. Genes like *C3ar1* and *Lgals3* (protein name galectin-3) are highly correlated with the transitional pseudotime, whose expression is gradually upregulated during the fate switch of ECs from quiescence to activation in fibrosis. Inhibition of galectin-3 via siRNA or protein antagonists in mice could alleviate the pathogenesis of IPF and the transition of ECs. With the stimulation of human pulmonary microvascular endothelial cells (HPMECs) by recombinant proteins and/or siRNAs for galectin-3 in vitro, β-catenin/GSK3β signaling and its upstream regulator AKT are perturbed, which indicates they mediate the EndMT progress. These results suggest that EndMT is essential to IPF process and provide potential therapeutic targets for vascular remodeling.

## Introduction

Idiopathic pulmonary fibrosis (IPF) is a chronic and progressive lung disease with few clinical treatment options^1^. It is characterized by the deposition of extracellular matrix (ECM) material in fibroblast foci, which may lead to the loss of lung compliance and disruption of gas exchange^2^. Recent evidence suggests that the origin of lung fibroblasts plays a central role in the development of pulmonary fibrosis^3,4^, which provides potential therapeutic targets and novel ideas for treatment strategies. Among the identified sources of fibroblasts, the endothelial-to-mesenchymal transition (EndMT)^5–7^ has been documented to occur in IPF, whereby lung endothelial cells (ECs) can transform into significant numbers of fibroblasts. This finding offers an explanation for the vascular regression that may be a result of the loss of ECs via EndMT, thereby contributing to the fibroblastic elements in these foci^8^. However, exactly how ECs undergo EndMT and the underlying molecular mechanisms remain incompletely understood, making it challenging to target vascular remodeling to ameliorate IPF.

The transition of a certain type of cells into another is usually characterized by the fine-tuning of different transcription factors, sequential expression of marker genes, and modification of cellular functions^9–11^. However, the transformation of ECs into the mesenchymal phenotype is not a simple binary event, which imposes great challenges for the pharmaceutical treatment of EndMT-related diseases. Recent studies attempted to define cell transformation during EndMT using single-cell RNA sequencing (scRNA-seq) approaches^12,13^, highlighting scRNA-seq as an important strategy for investing dynamic processes of cell transition. However, few studies aimed to develop therapeutic strategies or discussed the clinical relevance of their findings. Therefore, a deeper understanding of EndMT could be gained using scRNA-seq and bioinformatic tools, with more detailed portraits of pre-defined and possibly intermediate cell states in this process.

The current work aims to investigate EndMT as a dynamic process in IPF, identify key regulators of the transition process, and provide novel targets for the treatment of EndMT-related diseases such as IPF. We characterized heterogenous cell states during EndMT in a bleomycin (BLM)-induced IPF mouse model of IPF in single-cell resolution. Specifically, unsupervised pseudotemporal analysis^14–17^ abled us to reconstruct EndMT trajectory, discovering several genes that are highly correlated with the transitional pseudotime. These candidate genes were further validated for their significance using an in vitro model of TGF-β-induced EndMT, as well as the aforementioned IPF model in vivo. The results revealed that galectin-3 is a strong facilitator and an effective target for inhibiting EndMT and even IPF. Overall, this study provides a meaningful strategy to suppress EndMT and the progression of vascular remodeling during the development of IPF, with great potential for further translational research.

## Results

### Resident ECs decreased in BLM-induced pulmonary fibrosis

**Fig. 1:**
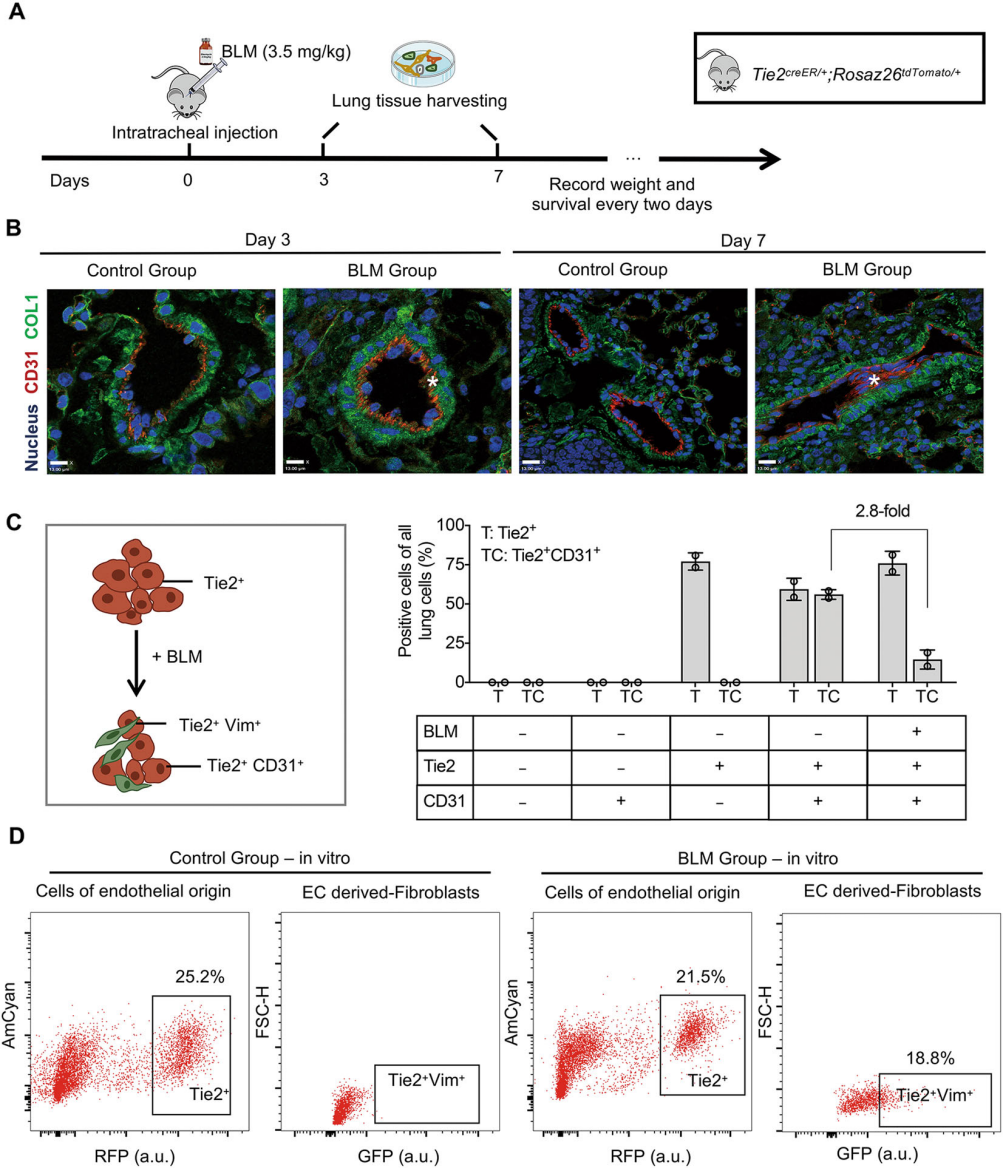
Verification of EndMT in a mouse model of pulmonary fibrosis. **A** Schematic diagram of the construction of the IPF model in *Tie2*^*creER/*^*+;Rosa26*^*tdTomato/+*^ mice based on a C57bl/6 genetic background. **B** Immunofluorescence imaging of ECs (*CD31*, red) and fibroblasts (COL1, green) in the BLM/control groups. The region in blue represents nucleus. Asterisks indicate the co-localization of ECs and fibroblasts. Scale bar = 13 μm. **C** Comparative analysis of the relative proportions of EC-derived cells (*Tie2*^+^) and ECs (*Tie2*^+^*CD31*^+^) in the BLM/control groups presented as means ± SD, *n* = 2. *x-axis:* different mice derived from C57BL/6. *Tie2*-positive cells represent all cells of endothelial origin; *Tie2* + *CD31* + cells represent resident ECs that remain endothelium-feature supplemented with BLM or saline solution. *y-axis:* the percentage of subpopulations of *Tie2*-positive and *Tie2CD31*-positive cells. *n* = 2. **D** Gating strategy for calculating the fraction that underwent EndMT (*Tie2*^+^Vim^+^) cultured in vitro for 72 h after isolation from lungs by PBS perfusion. Specifically, cells gated by rectangles in black are subpopulation of interest.

In order to investigate the prevalence of EndMT in pulmonary fibrosis, the pulmonary fibrosis model was established by intratracheal administration of BLM firstly. The overall design of EndMT tracing is shown in Fig. 1A. Following BLM treatment, the weight of the fibrosis model mice gradually decreased, accompanied by a considerable increase in mortality (Supplementary Fig. 1A, B). The deposition of collagen significantly increased and the lung architecture was severely damaged (Supplementary Fig. 1C–E).

To track the trajectory of the changes of ECs during fibrosis, we performed lineage analysis of fibroblasts using *Tie2*^*creER/*^*+;Rosa26*^*tdTomato/+*^ mice. Based on that, we used immunofluorescence staining to find that *CD31*-positive ECs scattered throughout the fibrotic area in the BLM-treated pulmonary fibrotic samples (BLM groups), while in the control groups *CD31* expression could only be detected in pulmonary microvascular and arterial blood vessels. Interestingly, a subset of the ECs in the BLM-induced pulmonary fibrosis groups was positive for the fibroblast marker COL1, while there was almost no immunoreactivity in the control groups (Fig. 1B).

At the same time, we performed flow cytometry to isolate *Tie2*^+^ cells (cells of endothelial origin) from normal and fibrotic lungs. The result showed that the subpopulation of *Tie2*^+^ *CD31*^+^ was decreased almost 2.8-fold in fibrotic lungs compared to that in the control group (Fig. 1C, Supplementary Fig. 2). Due to the complex nature of lung fibrosis in situ, we also extracted cells from lung tissues and cultured them in vitro to study their changes via FACS analysis. And the result showed that in the BLM group, there were appropriately 18% Tie2^+^Vimentin^+^ (which is the fibroblast special marker) ECs (Fig. 1D), which offers strong evidence that the number of ECs decreased and transitioned into fibroblasts during the pathological progression of pulmonary fibrosis.

### scRNA-seq reveals the transcriptional heterogeneity of ECs

**Fig. 2:**
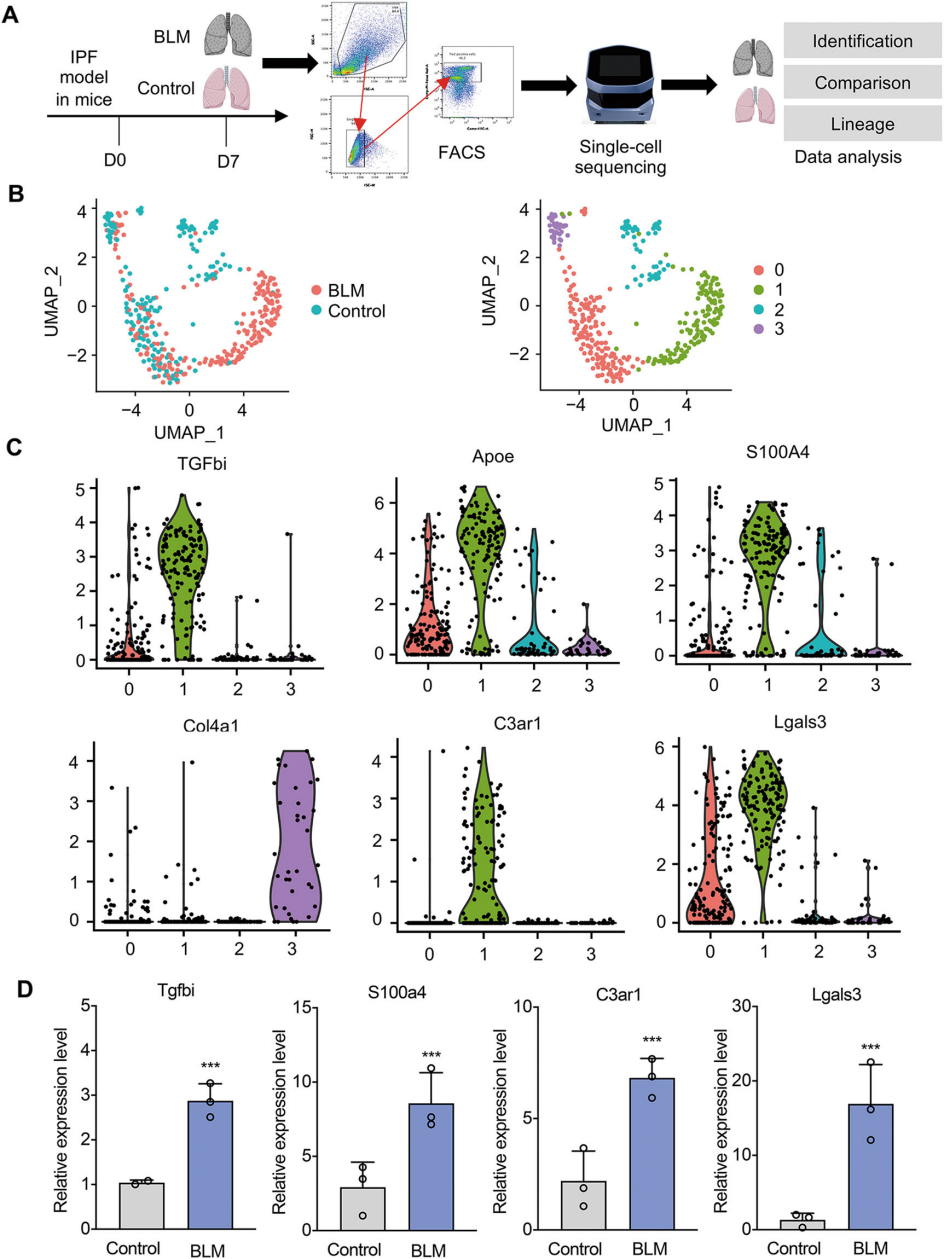
Transcriptional heterogeneity of ECs during EndMT in IPF at single-cell level. **A** Sing-cell transcriptome sequencing (scRNA-seq) using the Fluidigm C1 platform. Lung cells from the BLM and control groups were collected and sorted by FACS to harvest *Tie2*^+^ cells for scRNA-seq. **B** UMAP visualization of single-cell transcriptomes in the BLM and control groups. *Left panel:* colored by origin of cells (BLM or control). *Right panel:* colored according to the unsupervised clustering of cells. **C** Cluster-wide expression of EndMT-related markers/regulators screened among differentially expressed genes of high significance (*p* < 0.01) based on their GO-terms. **D** Expression levels of candidate regulators in whole lung samples from the BLM (day 7) and control groups according to RT-PCR. The data are presented as means ± SD, *n* = 3. *p < 0.05, **p < 0.01, ***p < 0.001.

To further explore the heterogeneity of ECs in PF, we identified subpopulations of the ECs that differ between normal and fibrotic lung tissues using scRNA-seq (Fig. 2A). After removing cells that expressed less than 100 transcripts, which indicates poor viability and likely apoptosis, a total of 391 single-cell datasets were obtained, among which there were 174 ECs from normal lungs (control) and 217 ECs from BLM-induced fibrotic lungs (BLM). Among the 38,720 coding genes in the mouse genome, 8350 were expressed by at least 5 cells in the control groups and 9497 in the BLM groups, while 9078 genes were expressed by at least 10 cells in both groups. UMAP visualization and clustering heatmap showed that ECs in the control group are well separated from ECs in the BLM group (Fig. 2B, Supplementary Fig. 3). After using gene-ontology (GO) analysis with the database from the Laboratory of Human Retrovirology and Immunoinformatics (LHRI, https://david.ncifcrf.gov/), we found that clusters which aggregated in the BLM group highly expressed genes related to inflammation (8.6%), oxidative stress (6.4%), and differentiation (4.6%) (cluster 1). Violin-maps of the four clustering markers within all EC states also showed several genes with very large heterogeneity compared to the saline-treated control group and BLM-treated pulmonary fibrosis group. The fibroblast-specific marker *S100a4* was obviously enriched in the fibrotic cluster (cluster 1), while *Col4a1*, which has been reported to be universally expressed in quiescent ECs^18,19^, was enriched in the saline-treated control cluster (cluster 3). Interestingly, *Tgfbi* (transforming growth factor, beta-induced), an ECM protein secreted by ECs that has been reported to be related to epithelial transformation and tumor metastasis^20,21^, was widely expressed in cluster 1 (Fig. 2C). Additionally, we were surprised to find that *C3ar1* (complement component 3a receptor 1) as well as *Lgals3* were significantly expressed in the fibrosis cluster. Moreover, the RT-PCR results confirmed that these genes identified in scRNA-seq were significantly expressed in the fibrotic groups (Fig. 2D). Taken together, the results indicated that ECs present heterogeneity in response to IPF, which may explain the activation of lung ECs in vivo.

### Reconstructing a transitional trajectory of ECs in IPF pathogenesis

**Fig. 3:**
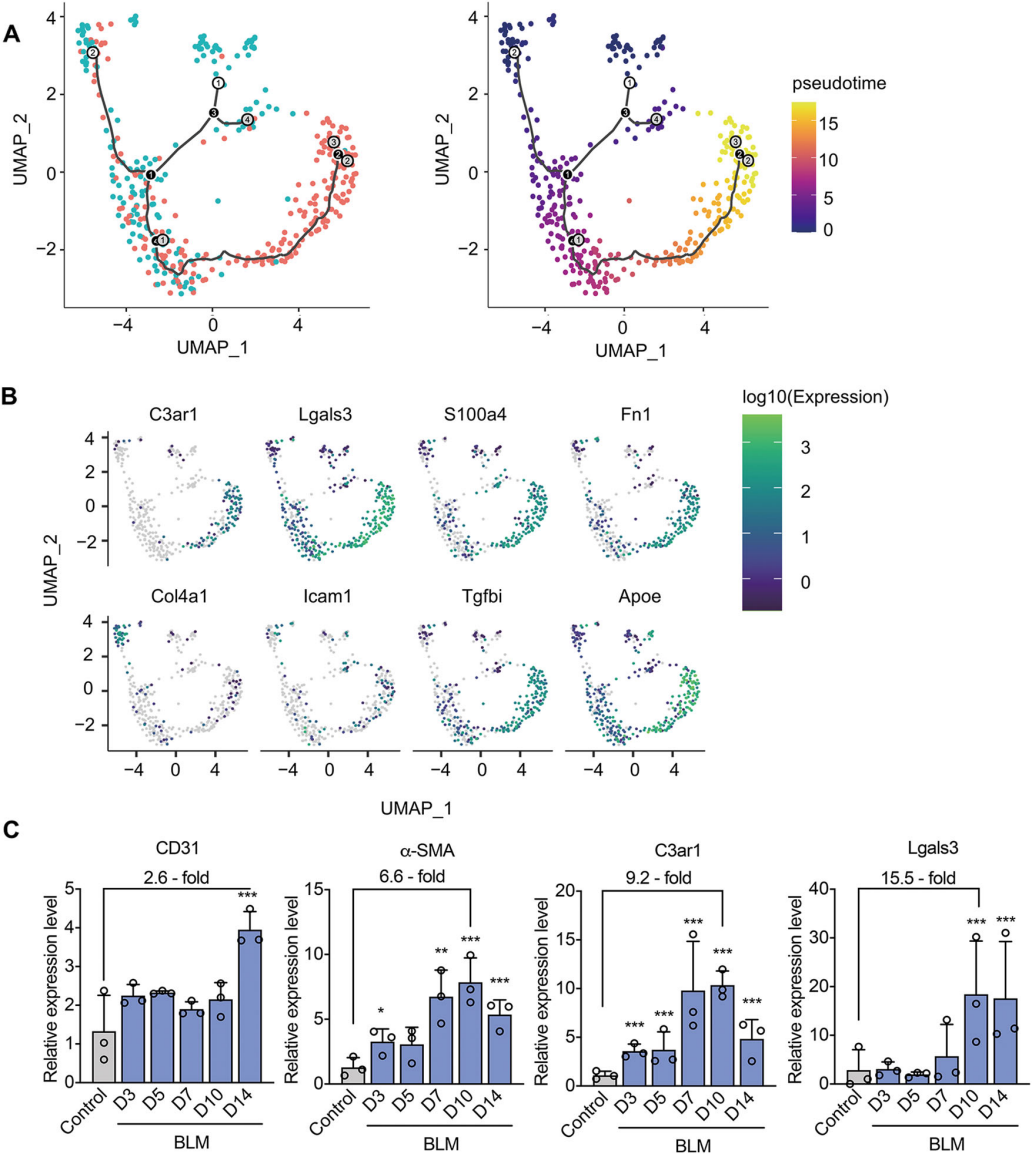
Pseudotemporal trajectory analysis of ECs revealing factors related to the EndMT process. **A** Merged pseudotime analysis of both groups revealing the transitional trajectory of ECs using Monocle3. Hollow circles: start/end nodes; black circles: branching nodes; black curve: pseudotemporal trajectory. **B** Expression of genes highly correlated with the pseudotemporal trajectory representing the EndMT process. *C3ar1* complementary component 3a receptor 1, *Lgals3* galectin-3, *S100A4* S100 calcium binding protein A4, *Fn1* fibronectin 1, *Icam-1* intercellular adhesion molecule-1, *Col4a1* collagen type IV alpha 1 chain, *Tgfbi* transforming growth factor beta induced, *Hif1a* hypoxia inducible factor 1 subunit alpha. **C** Expression levels of candidate regulators in whole-lung samples from the BLM and control groups according to RT-PCR. CD31: platelet and endothelial cell adhesion molecule 1, α-SMA: actin, alpha 2, smooth muscle, aorta. The data presented as the means ± SD, *n* = 3. *p < 0.05, **p < 0.01, ***p < 0.001.

To further depict the transcriptional dynamics of EC in PF, unsupervised pseudo-time reconstruction was applied using the R package Monocle 3^15^ to place ECs on a virtual time axis along which the cells are presumed to travel as they differentiate or become activated. Using this approach, cells from each cluster were added sequentially per time-point, and the data were visualized in terms of cellular states. The unsupervised pseudotime algorithm produced several branches and endpoints. As EndMT is expected to start from normal ECs, which then convert into a mesenchymal state over the course of fibrosis, we designated ECs from normal lungs (Fig. 3A, white points 1 and 2) as the starting points of the pseudotime. We also found that genes that are specifically activated in fibroblasts and ECs under stress conditions, such as *S100a4*, *Fn*1, and *Hif1a*, were highly expressed in the late stage of the pseudotime, as well as in the BLM group (Fig. 3B). Thus, it stands to reason that the pseudotime reflects the progression of EndMT.

We found that the two candidate genes *C3ar1* and *Lgals3* share highly similar expression trends with the fibroblast markers *S100a4* and *FN1* (Fig. 3C). According to previous literatures, both of them could stimulate fibrosis^22–24^, but few of them elucidate the dynamic tendency of these two mediators in the regulation of EC transformation in IPF. Thus, we used the RT-PCR method to quantify the expression of *C3ar1* and *Lgals3* over time in the BLM-induced IPF mice. At the same time, we measured the change trend of *CD31* and α-SMA, which is a specific marker of fibroblasts, as reference. Along the time axis of pulmonary fibrosis, the expression trends of *C3ar1* and *Lgals3* were highly consistent with the results of pseudo-temporal analysis (Fig. 3C). Moreover, the expression of these two genes was increased by approximately 10- to 15-fold compared to the control group. These results demonstrate that genes that play active roles in lung ECs during the fibrosis process are highly consistent with the predictions of pseudo-temporal kinetics. Furthermore, C3ar1 and galectin-3 may play critical roles in the dynamic transition of ECs leading to the emergence of fibroblasts.

### Inhibitors of C3ar1 and galectin-3 alleviate pulmonary fibrosis

**Fig. 4:**
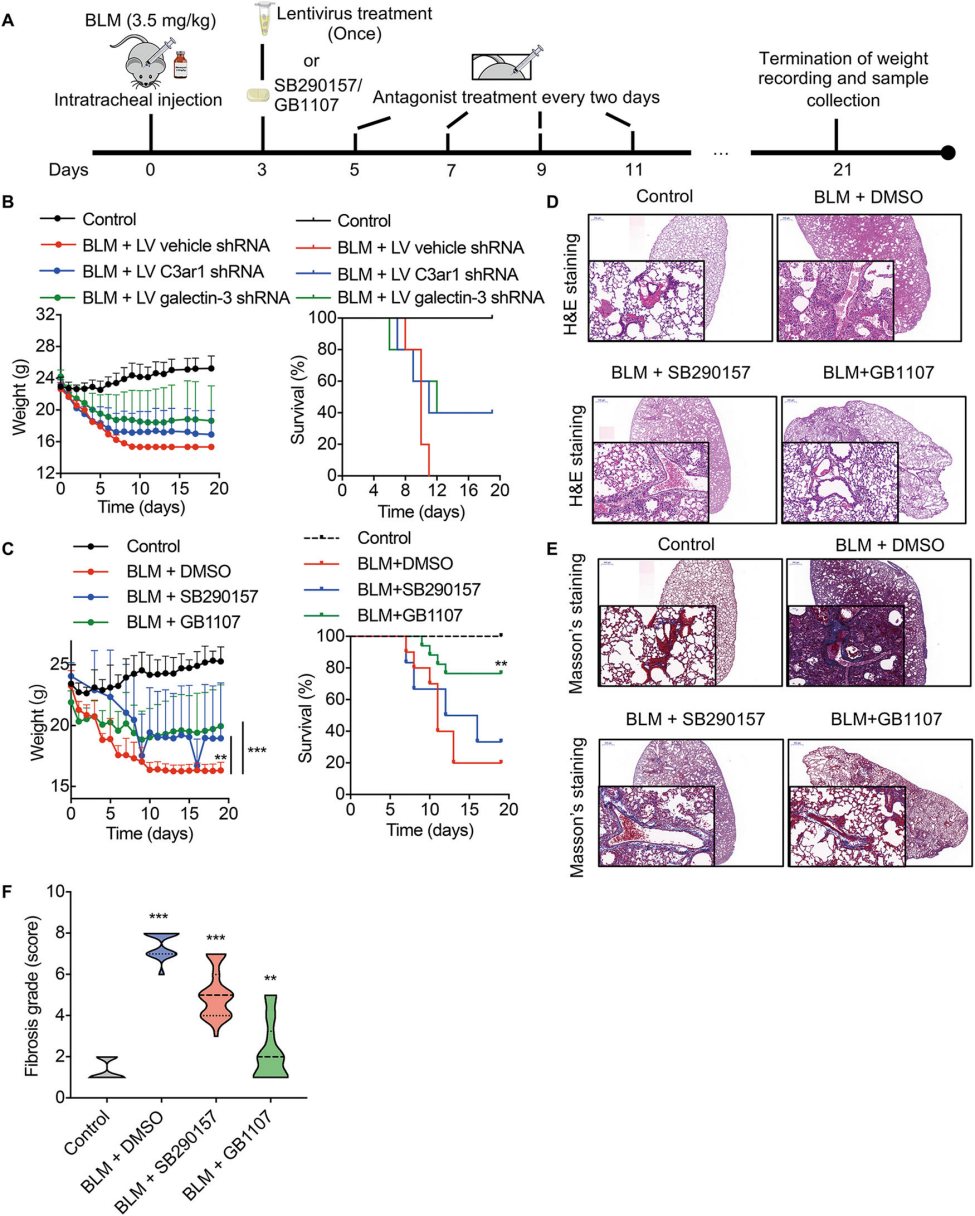
Alleviation of pulmonary fibrosis by targeting C3ar1 and galectin-3 in vivo. **A** Schematic diagram showing the functional exploration of C3ar1 and galectin-3 in IPF using lentivirus and galectin-3 interference (shRNA group), respectively. Alternatively, antagonists were used to block the binding affinity of C3a and galectin-3 (antagonist group). **B**, **C** Body weight and survival of IPF mice with galectin-3 shRNA lentivirus (**B**) (LV C3ar1 shRNA and LV galectin-3 shRNA, *n* = 5) or antagonists (**C**) (SB290157: antagonist of C3ar1; GB1107: antagonist of GB1107, *n* = 6). **D**, **E** HE and Masson’s trichrome staining of mouse lung sections following lentivirus or antagonist treatments. Scale bar = 500 μm (global view) or 50 μm (detailed view). **F** Semiquantitative morphological index/scoring of lung sections. The grade ranges from 1 (normal) to 8 (complete fibrosis). *n* = 30 for each group; *p < 0.05, **p < 0.01, ***p < 0.001.

To further confirm the positive role of C3ar1 and galectin-3 implied by the results of pseudotemporal-ordering analysis, we used lentivirus transfection and antagonists to interfere with their expression (Fig. 4A). The knockdown efficiency of C3ar1 and galectin-3 in CHO cells after transfection with the lentiviral plasmids (LV C3ar1 shRNA, LV galectin-3 shRNA and the control empty plasmid, LV vehicle shRNA) reached 91 and 52%, respectively (Supplementary Fig. 4). After nasal intubation to inject lentivirus into BLM-induced pulmonary fibrosis mice, fibrosis was alleviated, including a reduction of the weight loss and increase of the survival rate, which reached approximately 40% in the LV C3ar1 shRNA group and LV galectin-3 shRNA group, and was significantly higher than the survival rate of the sham group (LV vehicle shRNA) (Fig. 4B). At the same time, we intraperitoneally injected the mice with the C3ar1 antagonist SB290157 or the galectin-3 antagonist GB1107 during the development of BLM-induced pulmonary fibrosis. As shown in Fig. 4C, the tendency of weight change and survival rate was consistent with the lentiviral interference experiments. Notably, the survival rate after treatment with GB1107 reached 76%, compared to 33.3% in SB290157 group and 20% in the sham group (vehicle control with only DMSO).

The destruction of normal tissue architecture and ECM deposition was assessed using semi-quantitative grading of HE and Masson’s trichrome stained sections. As expected, GB1107 preserved the alveolar structure and reduced the deposition of ECM to a normal level. In the mice treated with SB290157, the intensity of the fibrotic areas and ECM deposition was more severe than in those treated with GB1107. At the same time, the alveolar thickness and ECM, especially collagen deposition, was clearly observed in the sham group which was treated with only the vehicle, DMSO (Fig. 4D–F). The immunofluorescence staining of lung tissues illustrated that the expression of C3a and galectin-3 was significantly reduced after antagonist treatment (Supplementary Fig. 5). Notably, we were able to observe that ECs widely express galectin-3 protein (co-labeled) in the BLM-induced pulmonary fibrosis group (BLM + DMSO), which corroborates the results of the single-cell analysis.

Taken together, these results demonstrate that inhibiting mediators screened by pseudotemporal analysis could alleviate the development of pulmonary fibrosis. Furthermore, inhibiting galectin-3 expression was more effective in alleviating fibrosis than inhibiting C3ar1.

### Galectin-3 stimulates EndMT in vitro

**Fig. 5:**
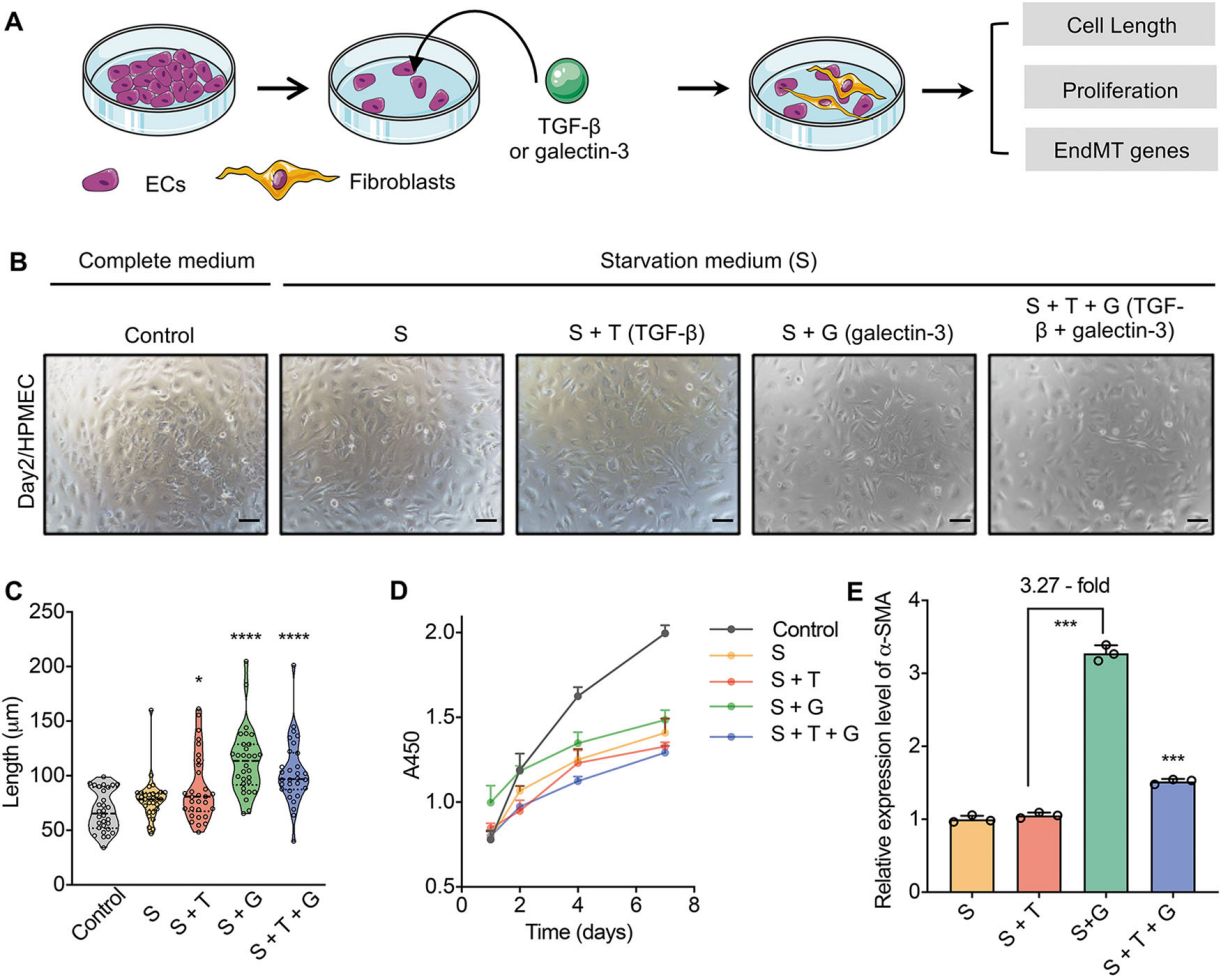
Galectin-3 promotes the EndMT process in vitro. **A** Schematic diagram showing the exploration of the potential of galectin-3 in inducing EndMT in vitro using HPMECs. **B** Morphological changes of HPMECs under different conditions. Complete medium (control): DMEM containing 10% FBS; starvation medium (S): DMEM containing 1% FBS; T: supplemented with 10 ng/ml TGF-β; G: supplemented with 1 μg/ml galectin-3. Scale bar = 50 μm. **C** Quantitative analysis of cell length of 30 randomly selected HPMECs from **B** using ImageJ software. **D** CCK-8 assay for determining the cytotoxicity of galectin-3 for ECs at days 1, 2, 4, and 7. **E** Expression levels of the fibroblast-related gene α-SMA in different experimental groups according to RT-PCR. Error bars represent standard deviations, *n* = 3. *p < 0.05, **p < 0.01, ***p < 0.001.

To further investigate if the role of galectin-3 in BLM-induced pulmonary fibrosis is related to EC activation and EndMT, we first used human lung micro-endothelial cells (HPMECs) to induce EndMT as described before^25–28^. We stimulated HPMECs with TGF-β as a positive control group, and additionally used recombinant galectin-3 protein to explore its function in EC activation. The morphology of ECs and the expression of classical markers, such as *CD31* and α-SMA, could be used to assess the occurrence of EndMT (Fig. 5A). With the performance of CCK-8, there was no significant effect of galectin-3 compared to the treatment with TGF-β, which means that galectin-3 has little cytotoxicity for HPMVECs (Fig. 5D). On the other hand, by statistically assessing the morphology of HPMECs at different time points, we found that they progressively changed from a normal cobblestone appearance into dispersed, spindle-shaped cells in the presence of TGF-β or galectin-3 (Fig. 5B, C, Supplementary Fig. 6). In addition, after measuring the expression of α-SMA, we found that α-SMA had a significant effect on the expression of galecin-3, which was upregulated 3.27-fold compared to the starvation group (Fig. 5E). Taken together, these results indicate that galectin-3 plays a vital role in the activation of ECs, and the results are highly consistent with the effects of TGF-β on the EndMT process.

### Galectin-3 promotes EndMT during IPF by activating the AKT/GSK3β/β-catenin signaling pathway

**Fig. 6:**
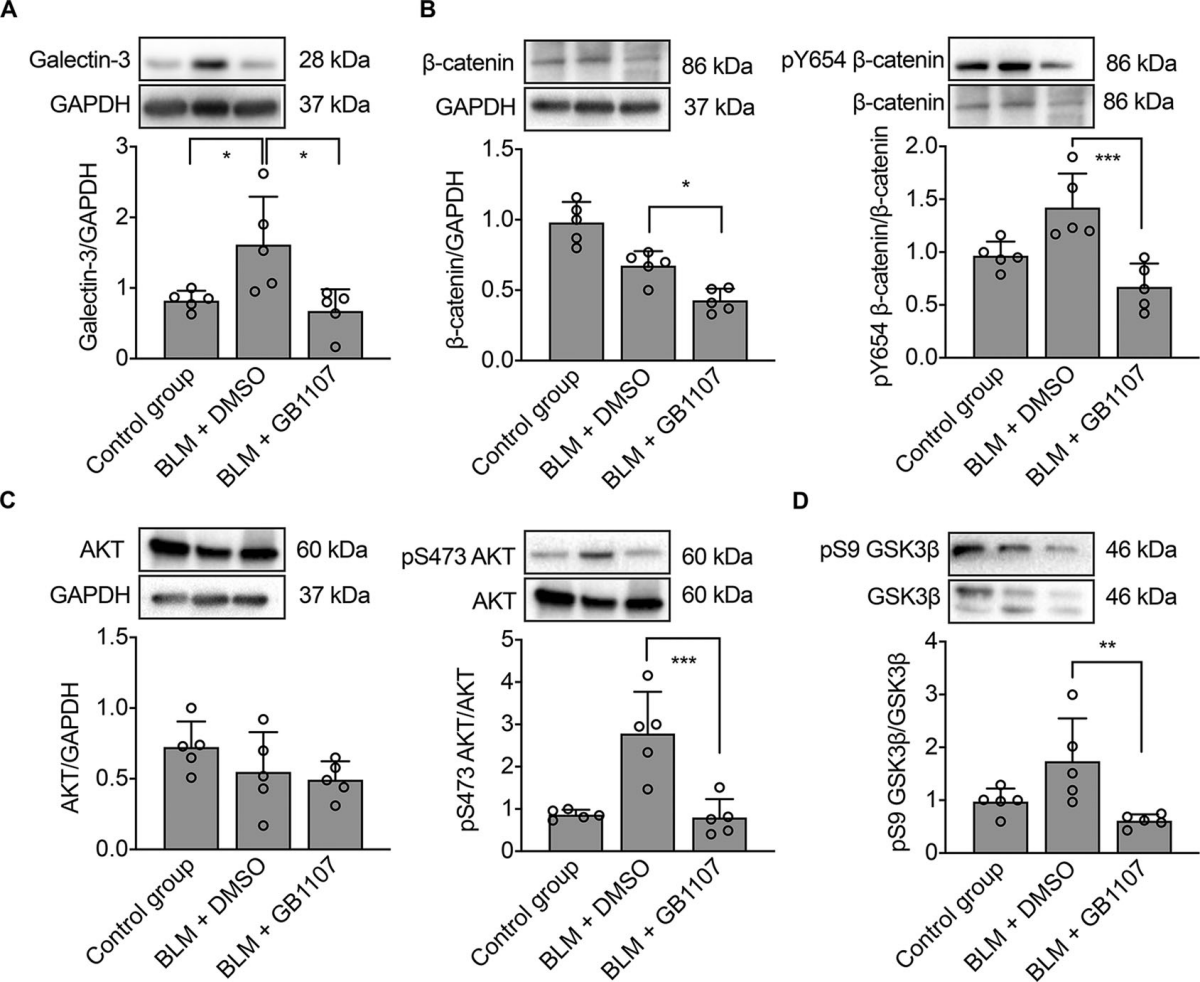
Inhibition of galectin-3 downregulates the AKT/GSK3β/β-catenin pathway in IPF mice. Western blot analysis of galectin-3 and AKT/GSK3β/β-catenin pathway proteins in lung tissue lysates. Control: saline for intratracheal injection. BLM + DMSO: positive control using BLM for pulmonary fibrosis model construction and intraperitoneal injection of vehicle (DMSO). BLM + GB1107: intraperitoneal injection of GB1107 to inhibit galectin-3 expression, *n* = 5 per group. Protein targets are **A** galectin-3; **B** β-catenin; pY654 β-catenin; **C** AKT, pS473 AKT; **D** pS9 GSK3β; *p < 0.05, **p < 0.01, ***p < 0.001.

β-catenin is the central player of the canonical WNT signaling pathway, which participates in many cellular process^28,29^. MacKinnon et al. reported that galectin-3 reduced the phosphorylation and nuclear translocation of β-catenin^30^, which indicated that galectin-3 may also impact the activation of ECs in BLM-induced pulmonary fibrosis. GSK3β, an upstream regulator of β-catenin, has been confirmed to be inhibited through phosphorylation by activated phospho-AKT^31^. In order to investigate how galectin-3 activates ECs to regulate vascular remodeling in BLM-induced IPF, we examined the protein expression of ECs and mesenchymal biomarkers along with regulators of the AKT/GSK3β/β-catenin singling pathway after treatment with the galectin-3 antagonist GB1107 or vehicle (DMSO). As can be seen in Fig. 6, the expression of galectin-3 was downregulated after treatment with the inhibitor GB1107 compared to the sham group (BLM + DMSO) and normal mice (control group). The level of total β-catenin in the GB1107 group was decreased compared to that in the BLM group, which was accompanied by a significant reduction of phosphorylated β-catenin (pY654 β-catenin, Fig. 6B), phosphorylated AKT (pS473 AKT, Fig. 6C), and phosphorylated GSK3β (pS9GSK3β, Fig. 6D). Therefore, it stands to reason that GB1107 inhibited galectin-3 expression and attenuated the development of BLM-induced pulmonary fibrosis by suppressing the AKT/GSK3β/β-catenin singling pathway.

**Fig. 7:**
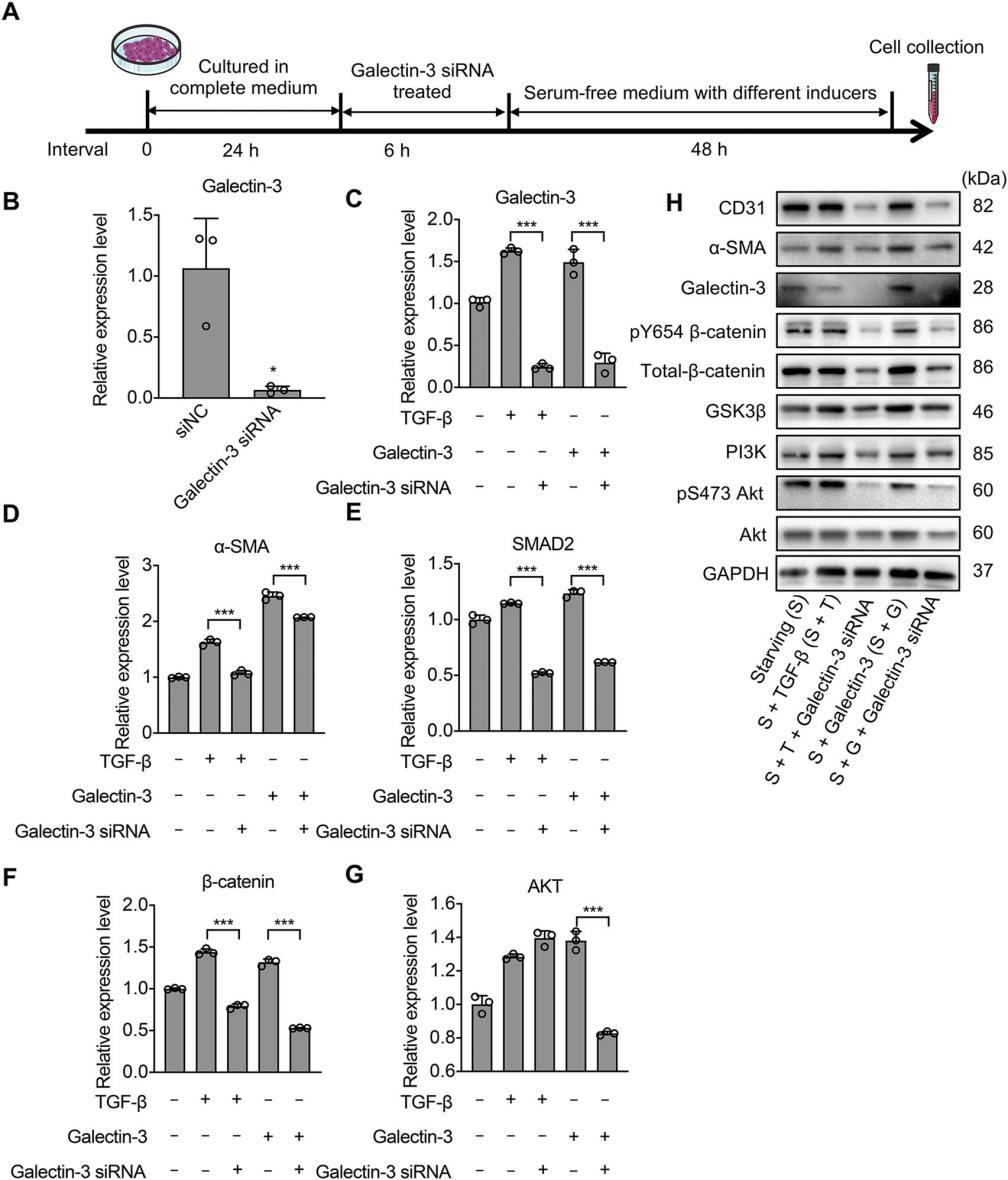
Inhibition of galectin-3 expression ameliorates the EndMT process by downregulating the AKT/β-catenin pathway in HPMECs. **A** Schematic showing the knockdown of galectin-3 expression with siRNA, and its impact on the transition of HPMECs. HPMECs were first cultured in complete medium for 24 h, and then transfected with siRNA targeting galectin-3 for 6 h. After treatment with the siRNA, the complete medium was replaced with starvation medium (DMEM containing 1% FBS), followed by further cultivation for 48 h. **B** The knockdown efficiency of galectin-3 siRNA according to RT-PCR. siNC: negative control. **C**–**G** HPMECs were stimulated with TGF-β (10 ng/ml, S + T) or galectin-3 (1 μg/ml, S + G) and interfered with galectin-3 siRNA (S + T + galectin-3 siRNA and S + G + galectin-3 siRNA, respectively) for 48 h. Analysis of the expression level analysis of the target genes galectin-3, β-catenin, and SMAD2, as well as the fibroblast-related genes α-SMA and S100A in different experimental groups using RT-PCR. Error bars represent standard deviations, *n* = 3. **H** HPMECs lysate was analyzed for CD31, α-SMA, and AKT/β-catenin signaling pathway regulators, total AKT and pS473 AKT, total-β-catenin and pY654 β-catenin, PI3K and GSK3β by Western blotting. *p < 0.05, **p < 0.01, ***p < 0.001.

Based on these results, we suspected that galectin-3 also plays vital roles in the activation and trans-differentiation of ECs. Therefore, HPMECs were treated with siRNA to inhibit the expression of galectin-3 and stimulated with the recombinant TGF-β or galectin-3 protein as described above (Fig. 7A). As expected, the expression of galectin-3 was almost completely suppressed by the siRNA (Fig. 7B). Additionally, galectin-3 expression was dramatically enhanced when the cells were treated with recombinant proteins TGF-β and galectin-3 without siRNA (Fig. 7C), which also indirectly indicated that EndMT could promote galectin-3 expression. At the same time, the biomarkers α-SMA and SMAD2, which act as regulators downstream of TGF-β^32^, along with the mediators AKT and β-catenin, showed notably decreased levels after galectin-3 siRNA interference compared to the untreated group (Fig. 7D–G). Additionally, Western blot analysis demonstrated that the level of phosphorylation of both β-catenin and AKT was also diminished following siRNA interference compared to that in the untreated groups (Fig. 7H). These results illustrated that galectin-3 activates the trans-differentiation of ECs via the AKT/GSK3β/β-catenin signaling pathway.

## Discussion

Only two drugs to date, Nintedanib and Pirfenidone, have been granted conditional recommendations for IPF, both of which attenuate fibrosis via anti-inflammatory effects. However, due to the limited understanding of the roles of blood vessels and vascular ECs in the pathogenesis of IPF, few studies attempted to inhibit the abnormal activation of ECs and consequent vascular remodeling. Analysis of the single-cell transcriptomes of ECs from normal/fibrotic lungs has enabled us to map the transitional trajectory of ECs for the first time. Several genes, including *Tgfbi*, *Apoe*, *Hif1a*, and *Nfkbia*, were found to be correlated with the pseudotemporal trajectory, although none as strongly as C3ar1 and galectin-3. Notably, these genes have also been reported as key regulators of IPF. For example, EndMT occurs in the early stage of radiation-induced pulmonary fibrosis and is dependent on *Hif1a* expression^33^. Their roles in EndMT should be further studied to identify possible new targets for the treatment of vascular remodeling in fibrosis, and such methods can also be used for other studies of cell-type transition processes.

Inhibiting the expression of galectin-3 showed a stronger effect against the development of pulmonary fibrosis than inhibiting C3ar1. It is possible that galectin-3 is expressed ubiquitously, not only in vascular ECs, but also in epithelial cells, macrophages, and myofibroblasts^34,35^, in which case its inhibition would have exerted a global and stronger effect against fibrosis. Furthermore, as an important member of the lectin family, galectin-3 stimulates the secretion of collagen and other ECM components, which are key features of fibrosis. Several studies have demonstrated that galectin-3 can stimulate EndMT in BLM-induced pulmonary fibrosis^30^, or lead to hepatic stellate cell activation in hepatic fibrosis^34^, offering new insights into fibroblast activation. Hence, it stands to reason that galectin-3 plays more vital roles in BLM-induced pulmonary fibrosis.

Our study proposes a new approach for studying the dynamic transition process of ECs and mapping EndMT states, provides important evidence for the transition of ECs and vascular remodeling during the fibrotic process, which has many strengths, but also has unique limitations. Firstly, we did not find completely novel markers that contribute to the EndMT process. Therefore, we hope to develop additional time-course analytic approaches to acquire more information on cell-type transition processes in the future. Secondly, it is well known that EndMT can also be induced by hypoxia or inflammatory factors in vivo, and it is challenging but important to prove that galectin-3 influences the progression of fibrotic disease mainly by inducing EndMT. Therefore, further research should be undertaken to establish conditional knockout models in order to track galecin-3 expression in ECs. Finally, based on the current research, we expect to be able to develop new combination therapy strategies based on small-molecule inhibitors of galectin-3, and propose new targets for translational medicine.

## Materials and methods

### Cell lines and treatment with recombinant protein

Human pulmonary microvascular endothelial cells (HPMECs, Beina Chuanglian Biotechnology, China) were cultured in DMEM-high glucose medium with FBS (10%), penicillin (100 IU), and streptomycin (100 μg/ml). HPMECs at passages 2–8 were used for further experiments.

Chinese hamster ovary (CHO) cells were a kind gift of the Shi Yan lab, Tsinghua University; CHO cells were cultured in DMEM-High glucose medium with FBS (10%), penicillin (100 IU), and streptomycin (100 μg/ml).

For EndMT induction in vitro, we used recombinant TGF-β (PeproTech, USA), C3a (Novoprotein, China), and galectin-3 (Cloud-clone, China) dissolved in dimethyl sulphoxide (DMSO, 1 mg/ml). For stimulation with the three factors, 50% confluent HPMECs were serum-starved (1% FBS) and incubated with TGF-β (10 ng/ml), C3ar1 (100 ng/ml), and galectin-3 (1 μg/ml) for 14 days. The culture medium was changed every two days. The control group was treated with complete medium or serum-starved medium.

All cells were maintained at 37 °C in an incubator with 5% CO_2_.

### Cell-counting kit assay

For the CCK-8 assay (Beyotime Biotechnology, China), HPMECs were seeded into 96 well plates and treated with different recombinant proteins. After culture for different lengths of time (1, 2, 4, and 7 days), the cells were incubated with 10 μl/well of the Cell Counting Kit-8 (CCK-8) solution for 2 h at 37 °C. The absorbance at 450 nm was measured using a conventional microplate reader.

### siRNA transfection

The siRNAs were purchased from GenePharma Corporation (Shanghai, China). The sequences are as follows: **siNC:** 5′-UUCUCCGAACGUGUCACGUTT-3′; **GAPDH siRNA**:5′-UGACCUCAACUACAUGGUUTT-3′; galectin-3 **siRNA**:5′-GCUCACUUGUUGCAGUACATT-3′; HPMECs were seeded into 10 cm culture dishes and incubated for 24 h. Then, the 70–80% confluent HPMECs were transfected with negative control group—siNC, positive control group—GAPDH siRNA and target galectin-3 siRNA using Lipofectamine 3000 (Thermo Fisher, USA) according to the manufacturer’s instructions. The final concentration of the siRNA solution was 50 nM.

### RNA isolation and quantitative RT-PCR

Total RNA was extracted from cells using TRIzol reagent (Invitrogen, USA) according to manufacturer’s instructions. Then, total RNA (1 μg) was reverse-transcribed using the StarScriptII First-strand cDNA Synthesis Mix with gDNA Remover (GenStar, China). The 2× RealStar Green Power Mixture with ROXII (GenStar China) was used to label the cDNA with fluorescent probes. Real-time-polymerase chain reaction (RT-PCR) was performed on a 7500 Fast Real-time PCR system (Thermo Fisher Scientific, USA), using the two-step RT-PCR program parameters provided by the manufacturer. The primers were designed using Primer Bank (https://pga.mgh.harvard.edu/ primerbank/) and NCBI (https://www.ncbi.nlm.nih.gov/) and the sequences are listed in Table S3.

Cycle threshold (ΔΔCt) values were calculated by normalization to GAPDH, and the gene expression levels were compared using the 2^−ΔΔ*Ct*^ values.

### Mice and fibrosis model

All wild-type (WT) and transgenic mice were based on the C57BL/6J background, aged 6 weeks at the beginning of experiments. WT mice were bred and housed at Tsinghua University Animal Facilities, with a 12 h light/dark cycle. *Tie2*^*creER/+*^*;Rosa26*^*tdTomato/+*^ mice were obtained and housed at the Shanghai Model Organisms Center, Inc. (Shanghai, China). Group allocation was randomized by Microsoft Excel (Microsoft Inc. USA) with no blinding.

To induce the pulmonary fibrosis model, the mice were anesthetized using tribromoethanol (350 mg/kg), and the lungs of the mice were treated with bleomycin (BLM) via intratracheal injection. BLM was prepared by dissolving sterile BLM sulfate powder (Ruitaibio, China) in sterile saline. BLM was administered via intratracheal injection (3.5 mg/kg in a total volume of 100 μl sterile saline)^36–39^. For the control group, the mice were treated with an equal volume of sterile saline.

### HE and Masson’s trichrome staining and scoring of lung sections

The lungs were perfused with phosphate buffer (PBS) from the right to the left ventricle of the heart. After perfusion, the lungs were surgically removed, the lobes separated, fixed in 4% paraformaldehyde (PFA), embedded in paraffin, and processed into 3 μm slices. The sections were stained with hematoxylin and eosin (HE) or subjected to Masson’s trichrome staining. Stained sections were scanned under a laser scanning confocal microscope (Zeiss, USA).

For semiquantitative morphological index (SMI) analysis, the lung sections were scanned using a 20× objective. Each captured field was individually assessed for severity of interstitial fibrosis and allotted a score between 0 and 8^40,41^.

### Immunofluorescence staining

For immunofluorescence staining, the tissue sections were placed in a repair box filled with EDTA antigen retrieval buffer (PH8.0) (Wuhan Servicebio Technology Co., China) for antigen retrieval in a microwave oven for 8 min. After retrieval, the sections were blocked with PBS (Solarbio, China) containing 5% fetal bovine serum (FBS, Thermo Fisher Scientific, Waltham, MA, USA) for 30 min at room temperature. Subsequently, the rat anti-mouse primary antibodies against *CD31* (1:100, BioLegend, CA, USA), COL1 (1:100, Servicebio, China), galectin-3 (1:100, Abcam, UK), and C3a (1:100, Abcam, UK) were incubated with the sample at 4 °C overnight. Then, an FITC-TSA (or CY3-TSA) conjugated secondary antibody was used to stain the samples for 10 min at room temperature in the dark. The stained sections were observed using a laser scanning confocal microscope (Zeiss, USA).

### FACS of cells of endothelial origin

The digested cells from lung tissues or cultured cells were respectively labeled with a flow cytometric antibody against FITC-CD31(1:100, BioLegend), FITC-CD90 (1:100, BioLegend), FITC-Vimentin (1:100, BioLegend), and APC-CY7-CD45 (1:100, BioLegend) and incubated at 37 °C for 10 min in the dark. Subsequently, the sections were washed with PBS containing 10% FBS. The cells were then analyzed via high-resolution flow cytometry (BD FACSAria, BD bioscience, USA).

For FACS data analysis, cells were sorted using 488 nm and 633 nm lasers, and the fluorescence signals were captured on FITC, CY-7, FSC (forward scatter), and SSC (sider scatter) channels. In order to ensure the viability of cells, 2000 events were recorded for each sample at a rate of 0.5 μl/s. Cytometry data were processed to generate the percentage values of positive events using FlowJo (version 10.4) software (Becton, Dickinson & Company, USA).

### Treatment with galectin-3 and C3a inhibitors in vivo

The galectin-3 inhibitor GB1107 (3,4-dichlorophenyl 3-deoxy-3-{4(3,4,5-trifluorophenyl)-1H-1,2,3-triazol-1-yl}-1-thio-α-D-galactopyranoside; *K*_d_ = 37 nM; MedChemExpress, USA) was prepared at a concentration of 2.5 mg/ml in 10% DMSO, 40% PEG300, 5% tween-80, and 45% saline. The C3a inhibitor SB290157 (N(2)-{(2,2-diphenylethoxy) acetyl}-L-arginine; IC_50_ = 200 nM; MedChemExpress, USA, 2.5 mg/ml) was prepared at 10% DMSO, 40% PEG300, 5% tween-80, and 45% saline.

For drug treatment, we first treated mice with BLM as described above. On the third day after model induction, we intraperitoneally injected the mice with SB290157 (0.1 mg/kg/day, *n* = 6) or GB1107 (0.1 mg/kg/day, *n* = 6) according to literatures^42,43^. These drugs were injected every other day until the weight of the mice fell below 15 g and were euthanized. The control group was treated with the vehicle (DMSO, *n* = 6).

### Lentivirus production and infection on mice

We firstly designed the core fragment of the shRNA lentivirus for C3ar1 and galectin-3 using the shRNA library of Tsinghua University. The detailed sequences are shown in Table S2. Then, HEK 293 T cells were transfected with the lentivirus packing plasmids PSPAX2 and PMD2.G at 90% confluence on the day of transfection. To assess the knockdown efficiency of the lentivirus, we transfected Chinese hamster ovary cells (CHO) when they reached about 70% confluence, using the lentiviral particles together with polybrene (Millipore, Sigma, USA) overnight. After 3 days, FACS was conducted to screen positive cells. Once the positive cells were collected, we used RT-PCR to measure the target gene expression.

For lentivirus infection (LV C3ar1 shRNA and LV galectin-3 shRNA), nasal intubation was adopted to deliver 1 × 10^8^ transducing units (TU) (20 μl per mouse, multiplicity of infection (MOI) = 10) into BLM-induced pulmonary fibrosis mice (*n* = 5 for each group) at the first day. For the control group, the empty lentivirus vector (LV vehicle shRNA) was injected into pulmonary fibrosis mice.

### Total protein extraction and Western blot analysis

Total protein from lung tissues (*n* = 5 for each group) and cultured HPMECs (*n* = 3 for each group) was extracted using lysis buffer (M-PER Mammalian protein Extraction Reagent, ThermoFisher, USA) with protease and phosphate inhibitors (ThermoFisher, USA). Protein concentrations were determined using the BCA protein assay (Yeasen, China). An equivalent amount of protein (20 μg) was subjected to 10% sodium dodecyl sulfate–polyacrylamide gel electrophoresis (SDS–PAGE) and transferred onto a PVDF membrane (0.45 μm, Merck Millipore, Germany). The membranes were blocked for 30 min with the NcmBlot blocking buffer (NCM Biotech, China) at room temperature and/or stripped with stripping buffer (CWBIO, China). The blocked membranes were individually incubated overnight at 4°C with rabbit anti-mouse (for lung samples) or mouse anti-human (for HUVEC samples) primary antibodies against CD31 (1:1000, Abcam, UK), α-SMA (1:100, Abcam, UK), β-catenin (1:1000, Abcam, UK), pY654 β-catenin (1:1000, Abcam, UK), GSK3β (1:5000, Abcam, UK), pS9 GSK3β (1:10000, Abcam, UK), galectin-3 (1:1000, CST, USA), PI3 kinase p85 alpha (1:1000, Abcam, UK), AKT (1:1000, CST, USA), pS473 AKT (1:2000, CST, USA), and GAPDH (1:10000, Abcam, UK). Then, the membranes were washed and incubated with secondary anti-rabbit or anti-mouse IgG antibodies (1:5000, CST, USA) for 1 h at 37 °C in immunoreaction enhancer solution (Toyobo, China). The chemiluminescence signal was detected using Enlight buffer (Engreen, China). The optical densities of the bands were quantified using Image Lab software (Bio-Rad, USA) and normalized to GAPDH as internal control.

### scRNA-seq of Tie2^+^ cells and data analysis

The healthy and BLM-treated (D7) *Tie2*^*creER/+*^*;Rosa26*^*tdTomato/+*^ mice *Tie2*^*creER/+*^*;Rosa26*^*tdTomato/+*^ mice (*n* = 2 for each group) were euthanized with CO_2_ and the lungs were thoroughly perfused with PBS to remove blood from the lung vascular beds^44^. The lungs were then removed from the thoracic cavity and cleared of extraneous tissue. The tissue samples were cut into small pieces and digested with 5 ml of Collagenase II (2.5 mg/ml, Invitrogen, USA) in a 50 ml tube for 30 min in a 37 °C water bath^45^. The resulting tissue/cell suspension was filtered through a 70 μm strainer and centrifuged for 5 min at 300 *g*. After removal of the supernatant, the cells were incubated with 1× Red Blood Cell Lysis Buffer (BioLegend, CA) for 5 min in ice. Then, the cells were subjected to FACS sorting to isolate tdTomato^+^ (*Tie2*^+^) cells.

scRNA-seq of the *Tie2*^+^ cells was performed on the 10–17 μm C1 HT IFC (Fluidigm, USA), immediately after the cells were collected by FACS. The cDNA library was sequenced on an HiSeq 2500 platform (Illumina, USA), followed by de-multiplexing using barcode sequences provided in the Fluidigm protocols. All downstream data analyses were conducted with R software. Principle component analysis (PCA), 2-dimentional data visualization by Uniform Manifold Approximation and Projection (UMAP)^46^ and cell clustering were realized using the Seurat3 package^47^, while the pseudotemporal trajectory and pseudotime-based differential expression analysis were performed using the Monocle3 package^14,48,49^.

### Statistical analysis

All samples were represented as biological replicates, and results are shown as means ± standard errors of the mean. GraphPad Prism 8 (GraphPad Software Inc. USA) was used to generate all charts and conduct all statistical analyses except the scRNA-seq results. One-way ANOVA followed by Tukey’s or Dunnett’s multiple comparison tests was applied to determine the significance of differences among different groups. Differences with *P* < 0.05 were considered statistically significant (*P < 0.05, **P < 0.01, ***P < 0.001).

Details of all reagents and products used in this study can be found in Table S1.

## Supplementary information

Supplemental Information

## Data Availability

The scRNA-seq dataset and the corresponding R code used in the present study are available at Github (https://github.com/THU-WQ-Lab/Mouse-PF-lung-Tie2-cell-scRNAseq).
