# Supplementary material for: Trajectory modeling of endothelial-to-mesenchymal transition reveals galectin-3 as a mediator in pulmonary fibrosis
Source: Cell Death Dis. 2021 Mar 26;12(4):327. doi: 10.1038/s41419-021-03603-0 (PMC7998015; doi:10.1038/s41419-021-03603-0)
Supplement: Supplementary file 1 — Supplemental Information [file 41419_2021_3603_MOESM1_ESM.docx]

**Supplementary Information:**

**Trajectory Modeling of Endothelial-to-Mesenchymal Transition Reveals Galectin-3 as a Mediator in Pulmonary Fibrosis**

**Authors:**

Wangyue Jia ^a,b,c,#^, Zhaoyan Wang ^a,b,c,#^, Ceshu Gao^d,#^ , Jian Wu ^d, *^, Qiong Wu^a,b,*^

**Affiliations:**

^a^ MOE Key Laboratory of Bioinformatics

^b^ Center for Synthetic and Systems Biology

^c^ Tsinghua-Peking Center for Life Sciences, Tsinghua University, Beijing 100084, China

^d^ Department of Neurology, Beijing Tsinghua Changgung Hospital, School of Clinical Medicine, Tsinghua University, Beijing, China.

^#^These authors contributed equally to this work.

^*^**Corresponding author:**

Jian Wu: wujianthu@mail.tsinghua.edu.cn

Department of Neurology, Beijing Tsinghua Changgung Hospital, School of Clinical Medicine, Tsinghua University, Beijing 100084, China

Qiong Wu: wuqiong@mail.tsinghua.edu.cn

School of Life Science, Tsinghua University, Beijing 100084, China

**Supplementary tables:**

**Table S1: Reagents used in this study**

| Item | Source | Identifier |
| --- | --- | --- |
| **Antibodies** |  |  |
| Alexa Fluor 488 anti-mouse CD31 | BioLegend | 102405 |
| APC-CY7 anti-mouse CD45 | BioLegend | 103116 |
| Alexa Fluor 488 anti-mouse CD90 | BioLegend | 105325 |
| Alexa Fluor 488 anti-mouse vimentin | BioLegend | 677809 |
| KLH-conjugated synthetic peptide corresponding to mouse CD31 | Servicebio | gb12063 |
| Anti-galectin-3 antibody | Abcam | ab76245 |
| KLH-conjugated synthetic peptide corresponding to mouse COL1A1 | Servicebio | gb11022-1 |
| HRP-conjugated mouse antibody | Servicebio | gb23301 |
| **Chemicals and Recombinant Proteins** |  |  |
| Bleomycin | Ruitaibio | B2434-2 |
| Phosphate buffer | Solarbio | P1020 |
| Dulbecco's Modified Eagle Medium Nutrient Mixture F-12 (DMEM/F12) | HyClone | SH30023.01 |
| Dulbecco's Modified Eagle Medium High-Glucose (DMEM-HG) | HyClone | SH30022.01 |
| Trypsin-EDTA 0.25% | HyClone | SH30042.01 |
| Trypsin-EDTA 0.05% | GIBCO | 25300062 |
| Penicillin-streptomycin | HyClone | SV30010 |
| Fetal bovine serum (FBS) | GIBCO | 10099141C |
| Collagenase II | Invitrogen | 17101-015-100 |
| RBC lysis buffer | BioLegend | 420301 |
| Human TGF-beta1-mammalian | PeproTech | 100-21-2 |
| complement component C3a/C3a | Novoprotein | CP21-10 |
| Recombinant galectin-3 (GAL3) | Cloud-Clone | RPA303Hu01 |
| Dimethyl sulfoxide (DMSO) | Solarbio | D8370-100 |
| 4% Paraformaldehyde (PFA) | Servicebio | G1101 |
| TRIzol | Invitrogen | 15596018 |
| SB290157 Trifluoroacetate; SB-290157 Trifluoroacetate | MedChemExpress | HY-101502A |
| GB1107 | MedChemExpress | HY-114409 |
| PEG300 | MedChemExpress | HY-Y0873 |
| M-PER Mammalian protein extraction reagent | Thermo Fisher | 7850 |
| Hal phosphatase inhibitor cocktail | Thermo Fisher | 78420 |
| Halt protease inhibitor cocktail, EDTA-Free | Thermo Fisher | 87785 |
| Lentivirus concentration reagent (10 × ) | BioGeek | BG20101L |
| **Commercial Assay Kits** |  |  |
| CCK-8 proliferation kit | Beyotime | C0038 |
| StarScriptII First-strand cDNA Synthesis Mix with gDNA Remover | GenStar | A224-10 |
| 2 × RealStar Green Power Mixture with RoxII | GenStar | A314-10 |
| TIANpure Midi Plasmid Kit | TIANGEN | DP108 |
| BCA Protein Quantification Kit | Yeasen | 20201ES76 |
| **Experimental Models: Organism/Strain** |  |  |
| Mouse:Tie2^Cre/+^ | Shanghai Model Organisms Center, Inc. |  |
| Mouse:Rosa26^tdTomato/+^ | Shanghai Model Organisms Center, Inc. |  |
| Human lung micro-endothelial cells (HLMEC) | Beina Chuanglian Biotechnology | BNCC337720 |
| **Software and Algorithms** |  |  |
| ImageJ | NIH |  |
| FlowJo software | FlowJo |  |
| GraphPad prism 8 | GraphPad |  |
| Confocal laser scanning microscope | Carl Zeiss |  |

| Item | Source | Identifier |
| --- | --- | --- |
| **Antibodies** |  |  |
| Alexa Fluor 488 anti-mouse CD31 | BioLegend | 102405 |
| APC-CY7 anti-mouse CD45 | BioLegend | 103116 |
| Alexa Fluor 488 anti-mouse CD90 | BioLegend | 105325 |
| Alexa Fluor 488 anti-mouse vimentin | BioLegend | 677809 |
| KLH-conjugated synthetic peptide corresponding to mouse CD31 | Servicebio | gb12063 |
| KLH-conjugated synthetic peptide corresponding to mouse COL1A1 | Servicebio | gb11022-1 |
| HRP-conjugated mouse antibody | Servicebio | gb23301 |
| **Chemicals and recombinant Proteins** |  |  |
| Bleomycin | Ruitaibio | B2434-2 |
| Dulbecco's Modified Eagle Medium Nutrient Mixture F-12 (DMEM/F12) | HyClone | SH30023.01 |
| Dulbecco's Modified Eagle Medium High Glucose (DMEM-HG) | HyClone | SH30022.01 |
| Trypsin-EDTA 0.05% | GIBCO | 25300062 |
| Penicillin-streptomycin | HyClone | SV30010 |
| Fetal bovine serum (FBS) | GIBCO | 10099141C |
| Collagenase II | Invitrogen | 17101-015 |
| RBC lysis buffer | BioLegend | 420301 |
| Human TGF-beta1 | PeproTech | 100-21-2 |
| Complement component C3a/C3a | Novoprotein | CP21-10 |
| Recombinant galectin-3 (GAL3) | Cloud-Clone | RPA303Hu01 |
| Dimethyl sulfoxide (DMSO) | Solarbio | D8370-100 |
| 4% Paraformaldehyde (PFA) | Servicebio | G1101 |
| TRIzol | Invitrogen | 15596018 |
| SB290157 Trifluoroacetate; | MedChemExpress | HY-101502A |
| GB1107 | MedChemExpress | HY-114409 |
| PEG300 | MedChemExpress | HY-Y0873 |
| **Commercial Assay Kits** |  |  |
| CCK-8 proliferation kit | Beyotime | C0038 |
| StarScriptII First-strand cDNA Synthesis Mix with gDNA Remover | GenStar | A224-10 |
| 2× RealStar Green Power Mixture with RoxII | GenStar | A314-10 |
| TIANpure Midi Plasmid Kit | TIANGEN | DP108 |
| **Experimental Models: Organism/Strain** |  |  |
| Mouse: Tie2^Cre/+^ | Shanghai Model Organisms Center, Inc. |  |
| Mouse: Rosa26^tdTomato/+^ | Shanghai Model Organisms Center, Inc. |  |
| Human lung micro-endothelial cells (HLMEC) | Beina Chuanglian Biotechnology | BNCC337720 |
| **Software and Algorithms** |  |  |
| ImageJ | NIH |  |
| FlowJo software | FlowJo |  |
| GraphPad prism 8 | GraphPad |  |
| Confocal laser scanning microscope | Carl Zeiss |  |

**Table S2: Oligonucleotides used for shRNAs.**

| **Oligonucleotide** | **Sequence** |
| --- | --- |
| homo-C3ar1 shRNA | CCGGGCAATCACATAGTGAAAGTTTCTCGAGAAACTTTCACTATGTGATTGCTTTTT |
| homo-Galectin-3 shRNA | CCGGCCCACGCTTCAATGAGAACAACTCGAGTTGTTCTCATTGAAGCGTGGGTTTTT |
| mouse-C3ar1 shRNA | CCGGCCCGTATTTGTATACCGTGATCTCGAGATCACGGTATACAAATACGGGTTTTT |
| mouse-Galectin-3 shRNA | CCGGCCCGCTTCAATGAGAACAACACTCGAGTGTTGTTCTCATTGAAGCGGGTTTTTG |

**Table S3: Primer sequences for RT-PCR**

| **Name** | **Sequence (5'-3')** |
| --- | --- |
| Homo-GAPDH-F | GGAGCGAGATCCCTCCAAAAT |
| Homo-GAPDH-R | GGCTGTTGTCATACTTCTCATGG |
| Mouse-GAPDH-F | AGGTCGGTGTGAACGGATTTG |
| Mouse-GAPDH-R | TGTAGACCATGTAGTTGAGGTCA |
| Homo-α-SMA-F | CTATGAGGGCTATGCCTTGCC |
| Homo-α-SMA-R | GCTCAGCCAGTAGTAACGAAGGA |
| Homo-S100A4-F | CGGGAGAAATTGCAGGAGGA |
| Homo-S100A4-R | AAGGTCAAGACGTGCCAGAG |
| Homo-ICAM1-F | ATGCCCAGACATCTGTGTCC |
| Homo-ICAM1-R | GGGGTCTCTATGCCCAACAA |
| Homo-COL1-F | GAGGGCCAAGACGAAGACATC |
| Homo-COL1-R | CAGATCACGTCATCGCACAAC |
| Homo-C3ar1-F | CCCTACGGCAGGTTCCTATG |
| Homo-C3ar1-R | GACAGCGATCCAGGCTAATGG |
| Homo-Galectin-3-F | ATGGCAGACAATTTTTCGCTCC |
| Homo-Galectin-3-R | GCCTGTCCAGGATAAGCCC |
| Homo-slug-F | CGAACTGGACACACATACAGTG |
| Homo-slug-R | CTGAGGATCTCTGGTTGTGGT |
| Mouse-Vimentin-F | CGTCCACACGCACCTACAG |
| Mouse-Vimentin-R | GGGGGATGAGGAATAGAGGCT |
| Mouse-C3ar1-F | TCGATGCTGACACCAATTCAA |
| Mouse-C3ar1-R | TCCCAATAGACAAGTGAGACCAA |
| Mouse-Galectin-3-F | AGACAGCTTTTCGCTTAACGA |
| Mouse-Galectin-3-R | GGGTAGGCACTAGGAGGAGC |
| Mouse-CD31-F | ACGCTGGTGCTCTATGCAAG |
| Mouse-CD31-R | TCAGTTGCTGCCCATTCATCA |
| Mouse-α-SMA-F | GTCCCAGACATCAGGGAGTAA |
| Mouse-α-SMA-R | TCGGATACTTCAGCGTCAGGA |
| Mouse-Hif1a-F | ACCTTCATCGGAAACTCCAAAG |
| Mouse-Hif1a-R | CTGTTAGGCTGGGAAAAGTTAGG |
| Mouse-NFkb-F | TGAAGGACGAGGAGTACGAGC |
| Mouse-NFkb-R | TTCGTGGATGATTGCCAAGTG |
| Mouse-S100A4-F | TGAGCAACTTGGACAGCAACA |
| Mouse-S100A4-R | TTCCGGGGTTCCTTATCTGGG |
| Mouse-Tgfbi-F | CAGCACGGCCCCAATGTAT |
| Mouse-Tgfbi-R | GGGACCTTTTCATATCCAGGACA |
| Mouse-Apoe-F | CTGACAGGATGCCTAGCCG |
| Mouse-Apoe-R | CGCAGGTAATCCCAGAAGC |

**Supplementary Figures:**

**
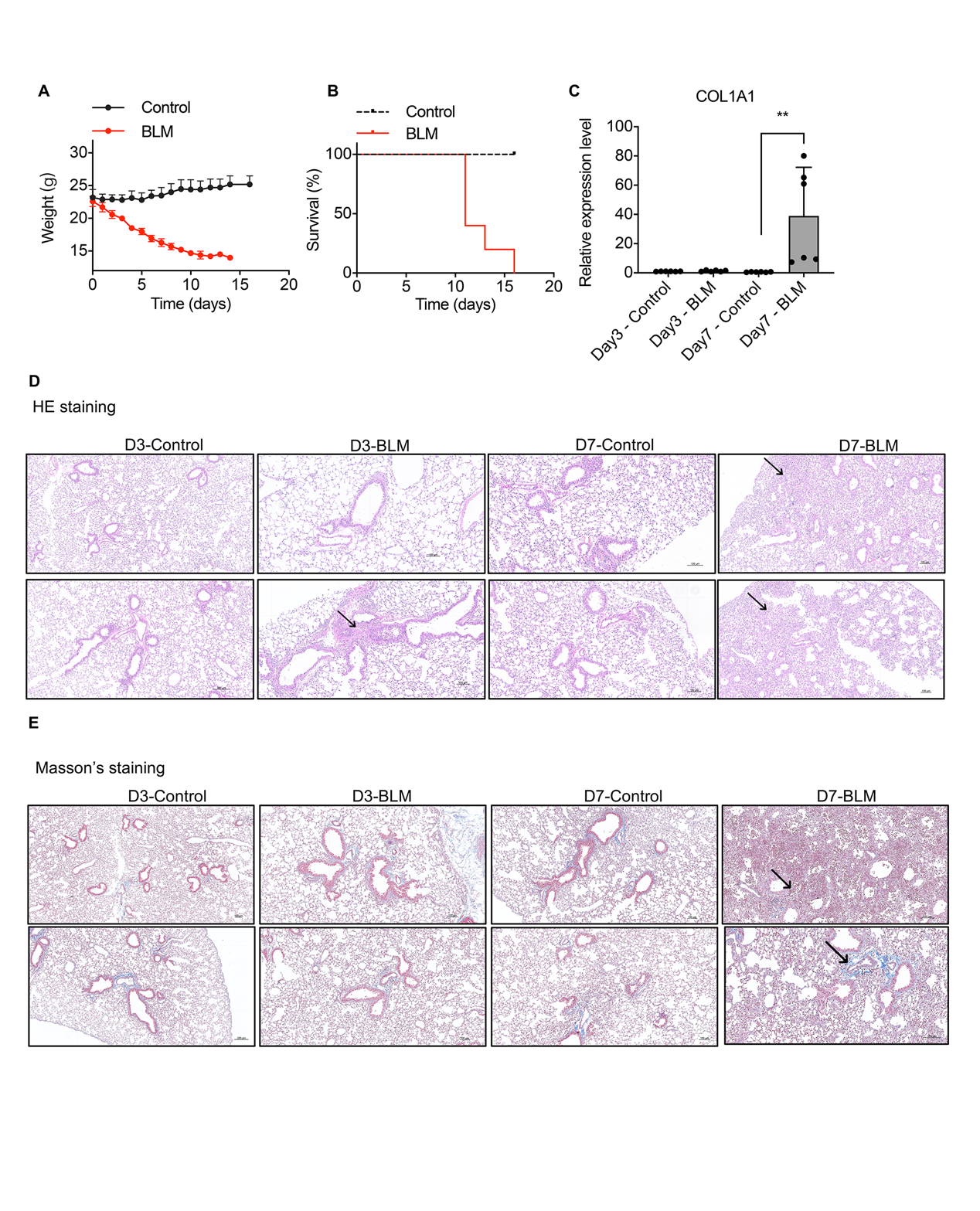
**

**Figure S1. Pathohistological assessment of BLM-induced pulmonary fibrosis.**

**A** The daily weight changes of mice. **B** The deaths in the BLM group on the 3rd (D3), 5th (D5), 7th (D7), 10th (D10), and 14th (D14) day were recorded for comparative analysis. n = 5. **C** The COL1A1 expression at D3 and D7 in BLM group. Control group: Mice treated with saline. *P<0.05, **P<0.01, ***P<0.001. **D**, **E** HE- and Masson-stained lung tissues. The lung tissues sampled on the 3rd and 7th day were analyzed by HE and Masson’s trichrome staining, respectively. The arrows in black indicate obvious fibrosis. Two images from 2 independent replicates of each group are shown. Scale bar = 100μm.

**
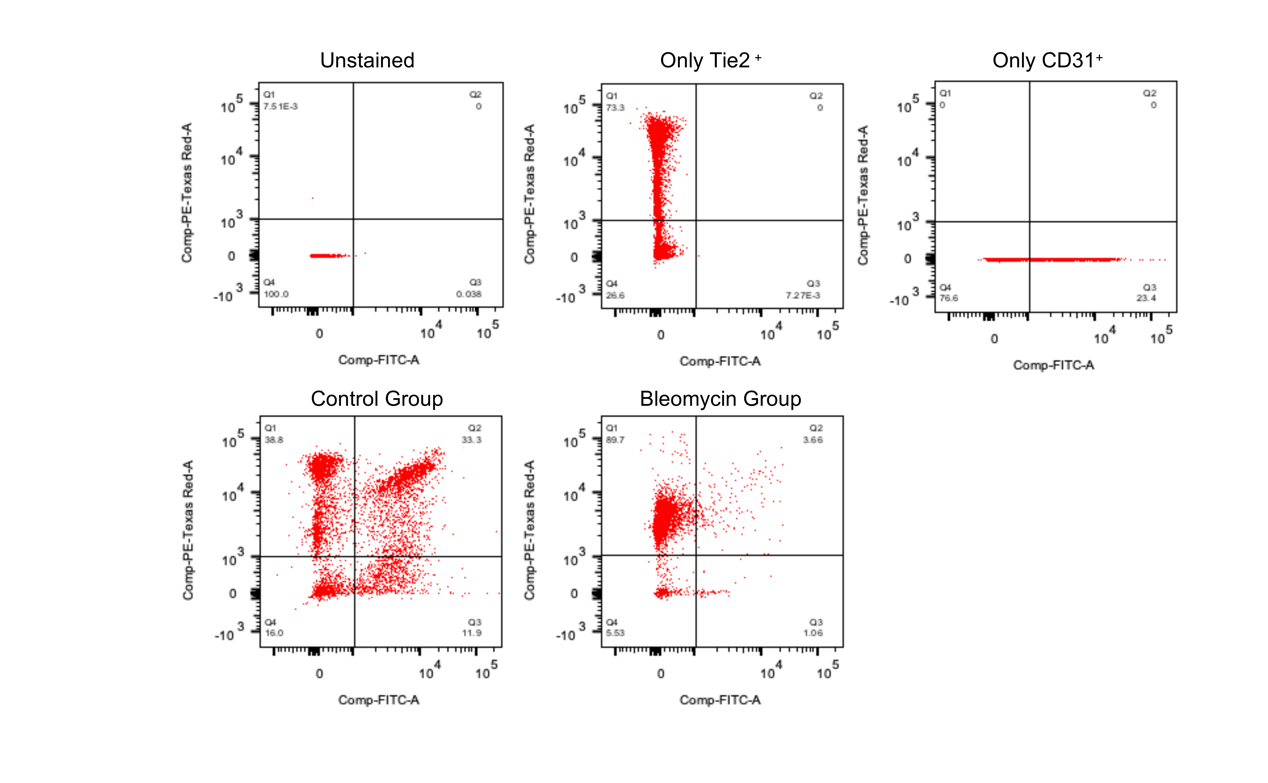
**

**Figure S2. Gating strategy of ECs and EC-derived fibroblasts.**

Cell counts harvested by FACS demonstrated that there was a significant decrease in the abundance of ECs in fibrotic lungs. Gate Q2 represents the remaining ECs after 14 days of induction with BLM.

**
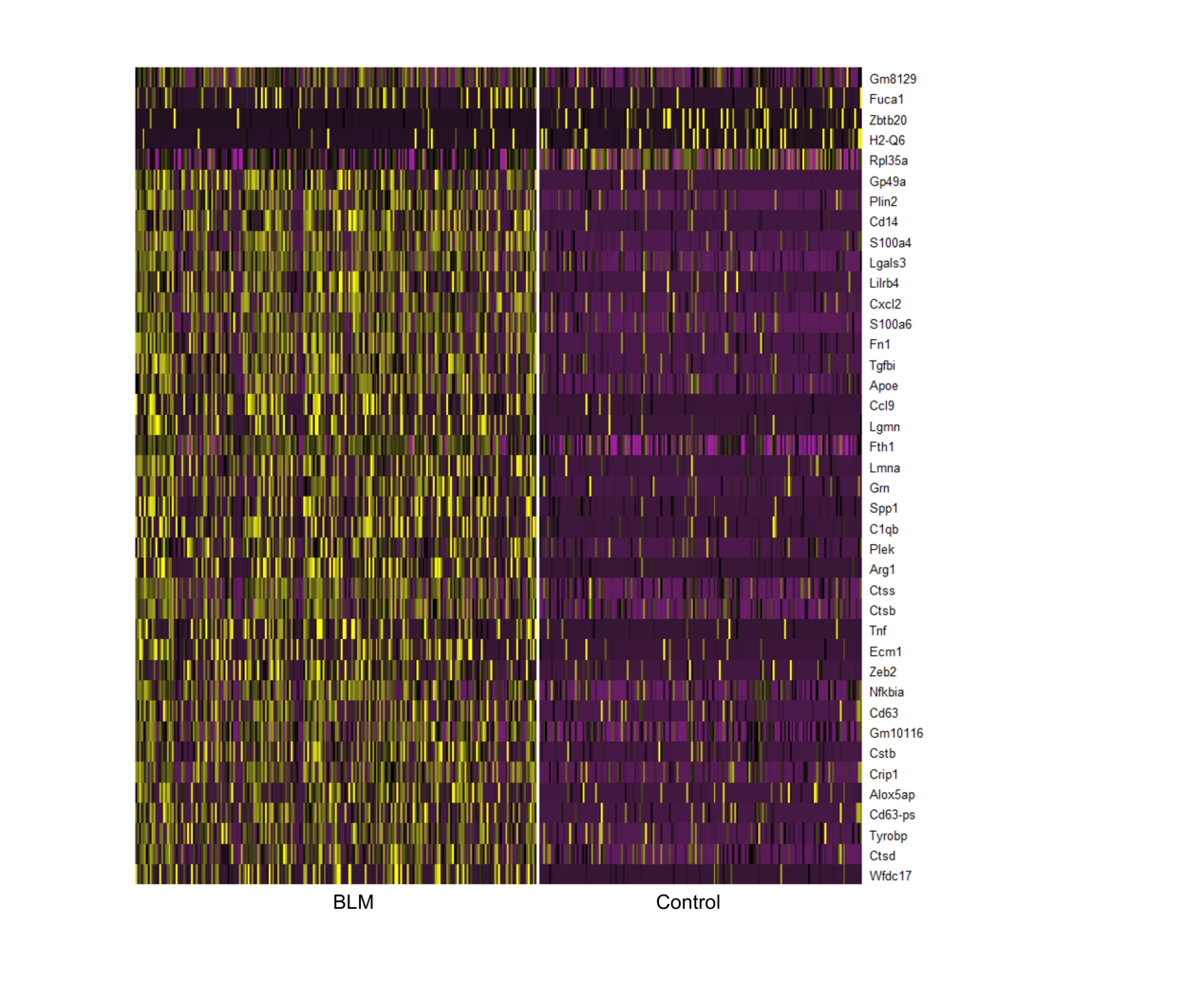
**

**Figure S3. Heatmap of gene clusters from fibrotic and normal lung tissues.**

Both rows of genes have been clustered. Yellow and purple represent the up- and down-regulation of target genes in fibrotic lung tissues (BLM) and normal lung tissues (NORM), respectively. *x-axis:* 217 independent ECs derived from the BLM group and 174 independent ECs derived from the control group (NORM). *y*-axis: genes with p-values of less than 0.01.

**
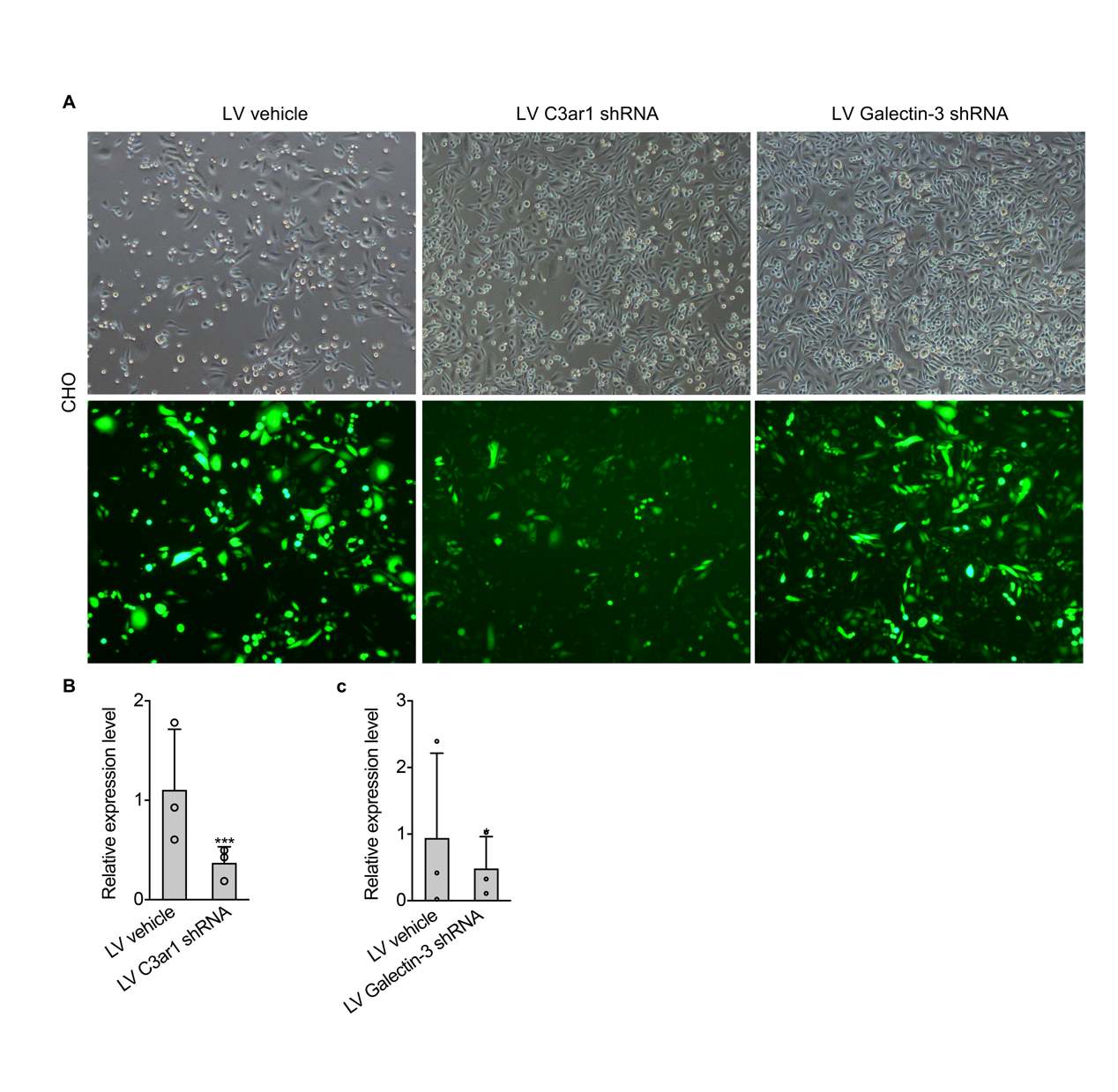
**

**Figure S4. CHO cells with gene knockdown screened according to immunofluorescence staining for specific markers.**

LV vehicle, LV C3ar1 shRNA and LV galectin-3 shRNA represent CHO cells transfected with short hairpin RNA of empty vector, C3ar1 and galectin-3, respectively. **A** Cells in green were successfully transfected with the shRNA plasmid. **B, C** The knockdown efficiency of C3ar1 and galectin-3 after transfection with lentivirus according to RT-PCR. Scale bar = 100μm. *P<0.05, **P<0.01, ***P<0.001

**
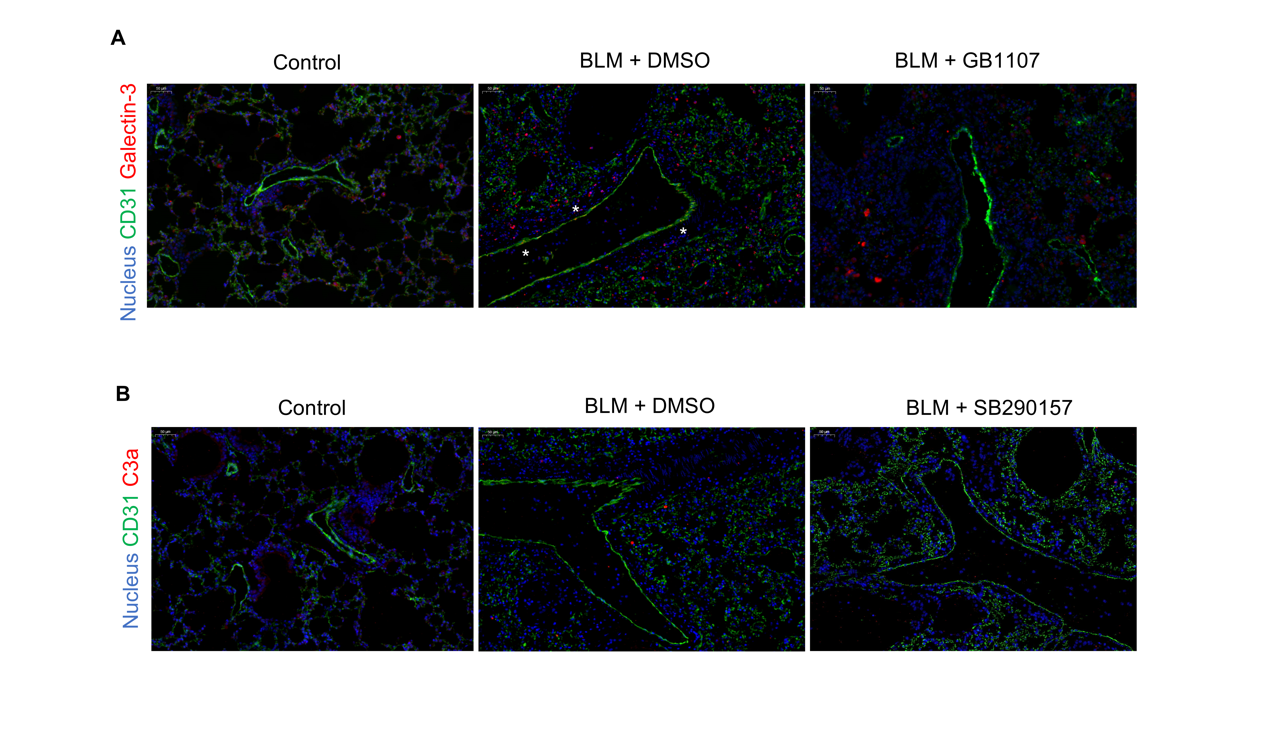
**

**Figure S5. Immunostaining for galectin-3 and C3a after treatment with their specific antagonists GB1107 and SB290157, respectively. A** Fluorescence staining including DAPI (nuclei), FITC (CD31) and tdTomato (galectin-3) in saline treated normal lungs (control), BLM-induced fibrotic lungs (BLM + DMSO) and BLM-induced fibrotic lungs with GB1107 treatment (BLM + GB1107); **B** Fluorescence staining including DAPI (nuclei), FITC (CD31) and tdTomato (C3a) in saline treated normal lungs (control), BLM-induced fibrotic lungs (BLM + DMSO) and BLM-induced fibrotic lungs with C3a antagonist (BLM + SB290157). Scale bar = 50μm.

**
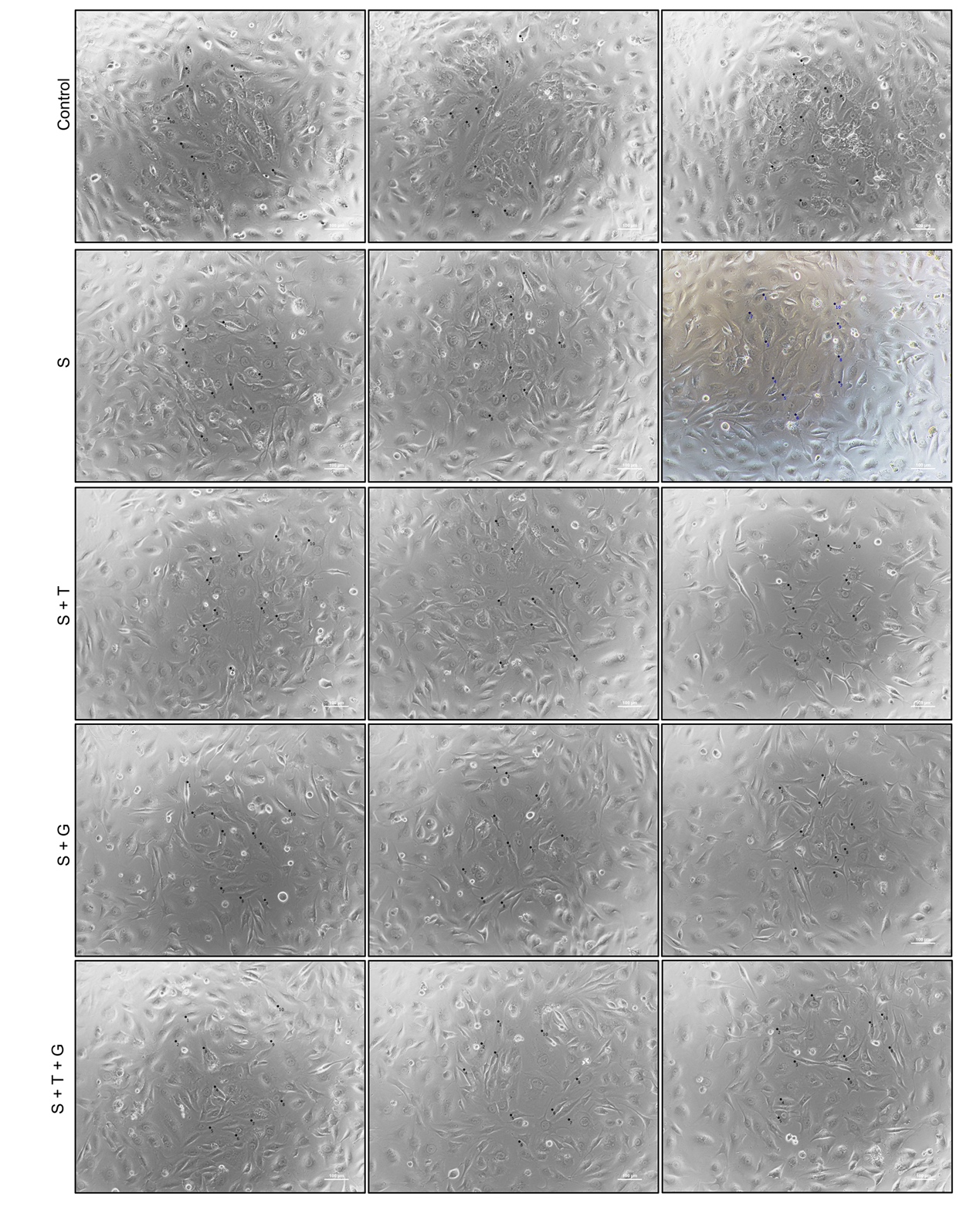
**

**Figure S6. Study of the morphology of ECs following treatment with C3ar1 and LGALS3.**

Morphological changes of ECs from different experimental groups. Three biological replicates were imaged by bright-field microscopy, and 30 cells of each group were randomly selected for cell length measurement (black dots indicate the selected cells). Scale bar = 100μm.
